# Validation of Inter-Subject Training for Hidden Markov Models Applied to Gait Phase Detection in Children with Cerebral Palsy

**DOI:** 10.3390/s150924514

**Published:** 2015-09-23

**Authors:** Juri Taborri, Emilia Scalona, Eduardo Palermo, Stefano Rossi, Paolo Cappa

**Affiliations:** 1 Department of Mechanical and Aerospace Engineering, Sapienza University of Roma, Via Eudossiana 18, I-00184 Roma, Italy; E-Mails: emilia.scalona@uniroma1.it (E.S.); eduardo.palermo@uniroma1.it (E.P.); paolo.cappa@uniroma1.it (P.C.); 2 Department of Economics and Management, Industrial Engineering (DEIM), University of Tuscia, Via del Paradiso 47, I-01100 Viterbo, Italy; E-Mail: stefano.rossi@unitus.it; 3 MAR Lab, Movement Analysis and Robotics Laboratory, Neurorehabilitation Division, IRCCS Children’s Hospital “Bambino Gesù”, Via Torre di Palidoro snc, I-00050 Fiumicino (RM), Italy

**Keywords:** Hidden Markov Model, inter-subject training, gait phase partitioning, Cerebral Palsy, Inertial Measurement Units, Wearable Sensor System, pediatric subjects

## Abstract

Gait-phase recognition is a necessary functionality to drive robotic rehabilitation devices for lower limbs. Hidden Markov Models (HMMs) represent a viable solution, but they need subject-specific training, making data processing very time-consuming. Here, we validated an inter-subject procedure to avoid the intra-subject one in two, four and six gait-phase models in pediatric subjects. The inter-subject procedure consists in the identification of a standardized parameter set to adapt the model to measurements. We tested the inter-subject procedure both on scalar and distributed classifiers. Ten healthy children and ten hemiplegic children, each equipped with two Inertial Measurement Units placed on shank and foot, were recruited. The sagittal component of angular velocity was recorded by gyroscopes while subjects performed four walking trials on a treadmill. The goodness of classifiers was evaluated with the Receiver Operating Characteristic. The results provided a goodness from good to optimum for all examined classifiers (0 < G < 0.6), with the best performance for the distributed classifier in two-phase recognition (G = 0.02). Differences were found among gait partitioning models, while no differences were found between training procedures with the exception of the shank classifier. Our results raise the possibility of avoiding subject-specific training in HMM for gait-phase recognition and its implementation to control exoskeletons for the pediatric population.

## 1. Introduction

Correct discrimination of gait phases during human walking represents a crucial issue in different research fields, such as in clinical assessment between normal and pathological gait [1,2], athlete coaching [3,4], monitoring health status through classification of daily life activities [5,6], human-machine interaction [7,8], and robotic rehabilitation [9,10].

The decision concerning the number of phases in the adopted partition model for the human gait varies according to the requirement of the specific research field. Even though the most widespread approach is based on the four-phase model [1,10,11,12], models with a different number of gait phases are also used [13,14,15]. Actually, Taborri *et al.* [13] found that a two-phase model is sufficient to control the knee module of an active orthosis where motor activation is needed only at the beginning of the stance and swing phases [14]. Moreover, for the goal of monitoring Parkinsons patient health status, the adoption of a six-phase gait model could be more appropriate [15].

To discriminate between gait phases, motion capture systems, with the integration of six-component force platforms, are considered the gold standard [16,17] even though they suffer from several limitations, such as soft issue artifacts, the inability to conduct analysis outdoors, and the necessity of a subjective evaluation of phase transition in gait models including more than four phases [18,19]. In order to overcome the previously cited limits, inertial sensors were extensively used in the last decade, due to their low cost, their wearability, and their efficiency in the recognition of gait patterns [20,21,22]. In particular, the angular velocities of lower limbs allowed researchers to discriminate between gait phases more accurately with respect to other inertial quantities, such as linear accelerations, due to greater peak-to-peak variability of angular velocity during gait [12,23]. As a consequence, gyroscopes are nowadays the most used sensors to feed algorithms for gait partitioning. The classification algorithms went through a developmental phase, evolving from a simple threshold-selection on raw data [24,25] to a machine-learning scheme [11,26,27]. Among the second group of algorithms, Mannini *et al.* reported that the ones based on Hidden Markov Models (HMM) demonstrated the best performance [11]. Moreover, a hierarchical weighted decision on data provided by different classifiers can be applied to improve the performance in the classification without increasing the computational load [10].

All classifiers based on a machine-learning scheme need a training procedure to optimally adapt model parameters to observed data [28]. The training is a subject-specific procedure that is very time-consuming in terms of data acquisition and processing. Furthermore, it often requires the integration of a larger set of sensors and the adoption of a more complex post-processing procedure for the acquisition of reference data. Several studies were conducted to bypass the subject-specific training, utilizing data gathered from a sample of subjects to train the HMM-based classifier. In particular, inter-subject training was performed on adults, both healthy [12] and with pathologies [29]. This approach substantially simplifies both the measurement system and the methodology, avoiding the time necessary to tune classifier parameters to the specific subject gait pattern. However, to the best of the authors’ knowledge, no studies have been conducted to implement an HMM that does not require a subject-specific training session for the gait-phase detection in typically developed children and in children with cerebral palsy. The need for such an evaluation stems from the higher variability of gait patterns in children with respect to adults, caused by an incomplete maturation of the ankle joint [30].

In an on-going research phase, we are developing an untethered wearable modular lower limb exoskeleton for children with motor disorders for daily domotic rehabilitation [31]. We plan to implement a simplified HMM classifier that does not require a preliminary trial for intra-subject training.

Thus, in this work, we test the validity of inter-subject training with the potential to bypass subject-specific training. In this context, the goal of the study is twofold. Firstly, we seek to validate the performance of this procedure with respect to the subject-specific training for both scalar and distributed classifiers based on data gathered from typically developed children and children with cerebral palsy. Secondly, we want to comparatively evaluate the goodness of the classifiers in the recognition of two, four and six phases.

## 2. Material and Methods

### 2.1. Theoretical Approach

In this study, we decided to apply two machine-learning algorithms, both based on the continuous Hidden Markov Model (cHMM), in order to compute gait phase recognition. More precisely, a scalar classifier and a distributed classifier, already proposed in the literature [1,10,12], were implemented. HMM is a statistical model widely utilized to estimate a sequence of hidden states in a time series [32] and it can be written as a set **λ** of parameters **A**, **π**, **w**, **µ** and **σ**:

(1) **λ** = (**A**, **π**, **w**, **µ**, **σ**)

In particular **A** represents the probability distribution matrix of state transition, **π** the initial state vector distribution, **w** a vector of mixture coefficients, **µ** and **σ** the means and standard deviations of the signal input of the model.

The scalar classifier (SC) processes one signal to generate a sequence of states. To be implemented, the initial values of two model parameters have to be chosen: **A** and **π**. Since we investigated three gait partitions, *i.e.* two, four and six phases, the number of possible hidden states to identify were, evidently, two, four and six. The two gait-phase model (2P) consists of stance phase (SP) and swing (SW). The four gait-phase model (4P) consists of flat foot (FF), heel off (HO), swing (SW) and heel strike (HS). Finally, the six gait-phase model (6P) consists of initial contact (IC), loading response (LR), mid stance (MS), terminal stance (TS), pre swing (PS) and swing (SW).

In all models, **A** is chosen as a left-right model, reflecting phase sequence in a normal gait, as already presented in Taborri *et al*. [10]; **π** is chosen, giving the same probability to all states, since the initial state of model is unknown. The use of an SC as a feature classifier usually requires performing two subsequent stages to optimize algorithm performance. The first stage includes the collection of a training data-set for computation of the model parameters obtained by means of the Baum-Welch algorithm [28], which is the most commonly used to solve the training problem. In the second stage, the estimation of the most likely phase sequence is performed using a modified version of the Viterbi algorithm [1,28] in order to reduce the computational load and provide real-time gait detection. In particular, outputs of training procedure for each model with *j* hidden states are:

- The trained probability distribution matrix of state transition **A**^tr^;
- The trained vector of mean values **µ**^tr^;
- The trained vector of standard deviations **σ**^tr^;
- The trained vector of mixture coefficient **w**_j_^tr^.

The outputs of the second stage, that is, the validation procedure, consisted of the most likely sequence of states **L**^SC^ and the probability associated to each state *γ_j_*.

The distributed classifier (DC) instead is based on a weighted hierarchical decision and performs the processing of two or more **L**^SC^ according to the algorithm proposed by the authors of the present paper [10] to obtain the final likely sequence **L**^DC^. The implementation of a DC is based on the choice of the number of simultaneous signals to be processed and of the distributed transition matrix **A**^DC^. In particular, we selected two signals and a right-left-right **A**^DC^. More details on the SCs and DCs can be found in [11,28] and [10], respectively.

### 2.2. Participants

Twenty children were recruited in the experimental procedure. In particular, a control group of ten typically developed children (TD, 9.5 ± 2.0 years) and an experimental group with ten children with hemiplegia (HC, 7.8 ± 2.8 years) were examined. The HC group was recruited among the patients of the Pediatric Neuro-Rehabilitation Division of the IRCCS Children’s Hospital “Bambino Gesù” (Rome, Italy). The entire experimental protocol was approved by the Ethics and Medical Board of the IRCCS Children’s Hospital “Bambino Gesù”. Typically developed children were involved if: (i) they had no known pathologies affecting their gait and (ii) they were age-matched with pathological children [33]. Subjects with cerebral palsy were included if: (i) they were able to perform a walking task without the assistance of a device and (ii) they presented hemiparesis or diparesis, congenital or acquired, excluding ones in which the acute event occurred in the last six months. The purpose of the study and the experimental methodologies used in the study were explained to the parents, oral informed consent was obtained from participants and written informed consent was obtained from participants’ parents.

### 2.3. Experimental Procedure

**Figure 1:**
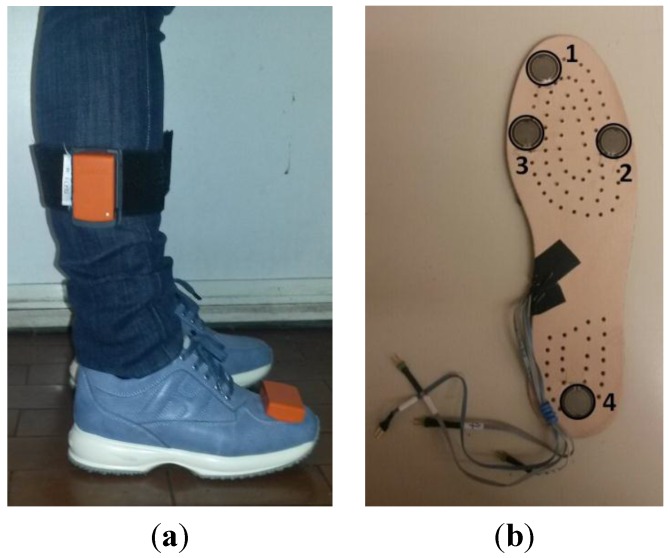
Sensor positioning on the subject. (**a**) Position of IMUs on participants’ lower limb; (**b**) position of foot-switches under participant’s instrumented foot (1: toe, 2: fifth metatarsophalangeal, 3: first metatarsophalangeal and 4: heel).

All subjects were instrumented with two IMUs (Xbus Master MTx, Xsens Technologies, Enschede, The Netherlands) placed on foot and shank of the dominant leg for TD and on the more affected leg for HC. By means of the gyroscopes embedded into the two IMUs, angular velocities of the body segments in the sagittal plane were acquired. In addition, four foot-switches (FSRs, Wave Cometa, Milan, Italy) were embedded in the sole and positioned under the toe, the first and the fifth metatarsophalangeal articulations and under the heel of the foot. The output of foot-switches allowed us to evaluate the contact between foot and ground. Thus, a sequence of gait phases, considered a reference signal, was obtainable during the gait. The accuracy of the footswitches’ outputs as reference signals was evaluated by means of static trials conducted prior to the dynamic ones, in which subjects were asked to place feet on the ground in different positions to verify the precision in the on/off activation of the footswitches. Correct discrimination was shown in all the three models. Raw data were gathered by foot switches at 200 Hz, while gyroscope data were sampled at 50 Hz. The stabilization obtainable via implementation of Kalman filter, to limit the effects induced by indoor magnetic distortions [34] to a reasonable level, was unnecessary as we gathered only gyroscope data from the set of sensor outputs provided by the IMU sensors. In order to align one of the sensible axes of the IMUs with the sagittal axis of each body segment, we performed a manual alignment procedure, consisting of an expert operator precisely positioning the sensors on the subject. The methodology is easier to perform than a functional calibration procedure to facilitate the analysis of children with pathological gait [35,36,37] and it allows us to obtain better results when only one axis for each sensor has to be acquired. Figure 1 shows the positions of IMUs and foot-switches on the subject.

Subjects were asked to walk on a treadmill with inclination 0% (Level walking—L) and 8% (Incline walking—I) at two speeds, *i.e.*, 1.0 km/h (L1.0 and I1.0) and 1.5 km/h (L1.5 and I1.5) for at least 60 s. Each walking condition was repeated twice. Participants were tested individually. Instrumentation procedure and walking tasks covered a duration of approximately 30 min. TD did not express fatigue during the experiment, while HC rested between each condition.

### 2.4. Data Processing

Acquired data were processed off-line using MATLAB software (MathWorks, 2012b, Natick, MA, USA). Angular velocity signals were treated with a low-pass Butterworth filter with a cut-off frequency of 15 Hz. To quantify the sensitivity to IMU position we conducted a preliminary simulation by imposing three different rotations to the angular velocity vector acquired during one trial: (i) 2° around frontal axis; (ii) 2° around transversal axis and (iii) a rotation of 2° around both frontal and transversal axes. Then, we compared results obtained using the new sagittal component of angular velocity with the reference one in terms of G values. The maximum decrease of G is approximately equal to 0.02. This finding implies that the cross-talk between sagittal and coronal/transverse kinematics can be considered negligible for the purpose of the present experimental analysis. Then, gyro signals were partitioned into gait phases, according to the particular model, by means of foot-switch data. The partitioning logic for the three examined models, based on pressed foot-switches, is shown in Table 1.

**Table 1:** Combination of active foot-switches to partition the gait cycle. Logic of partitioning in the two-phase model, 2P, four-phase model, 4P, and six-phase model, 6P. FF is the flat foot, HO the heel off, HS the heel strike, IC the initial contact, LR the loading response, MS the mid stance, PS the pre swing, SP the stance phase, SW the swing and TS the terminal stance. (x) indicates that foot-switches are pressed at the same time, and (*) indicates that at least one of the foot-switches marked are pressed.

Foot-Switch	2P		4P				6P					
	SP	SW	FF	HO	SW	HS	IC	LR	MS	TS	PS	SW
Heel	*		x			x	x	x	x			
5th metatarsus	*		x	*				x	x	x		
1st metatarsus	*		x	*					x	x		
Toe	*		x	*							x	

**Figure 2:**
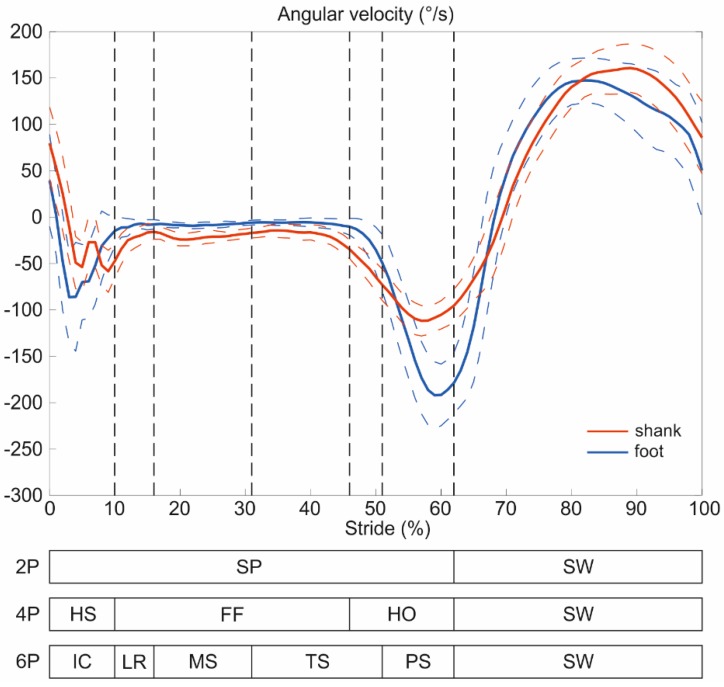
Angular velocities of shank (red) and foot (blue) partitioned into two, four and six phases, relative to healthy children. FF is the flat foot, HO the heel off, HS the heel strike, IC the initial contact, LR the loading response, MS the mid stance, PS the pre swing, SP the stance phase, SW the swing and TS the terminal stance.

Later, the partitioned angular velocity data of each phase were normalized in time length. Mean and standard deviation of the obtained dataset for each phase were calculated and used in the training stage of the HMM models, *i.e.*, **µ** and **σ** of Equation (1). An example of angular velocity signals of shank and foot divided into two, four and six gait phases, relative to healthy children, is reported in Figure 2.

In order to train the Hidden Markov Model, two different procedures were conducted: (i) the typical intra-subject procedure, here addressed as subject-specific training (SST); and (ii) the inter-subject procedure here addressed as standardized parameters training (SPT).

The SST model parameters for each subject were obtained by means of the angular velocity data measured during the first repetition of each walking condition. The SST model was then tested via the second repetition of the same condition. The cross-validation [1] was repeated for each subject of both groups, *i.e.*, TD and HC.

Since pathological gait presents a different pattern for each subject, we decided to construct the standardized parameters set with data gathered from healthy subjects; thus, SPT is a procedure based on a paradigmatic walking gait of healthy age-matched subjects. The SPT model parameters were obtained based on angular velocity gathered from the TD group during the first repetition of each walking condition. The angular velocity of the examined subject cannot be included in the training dataset, so we used a different approach for the two groups to validate the proposed procedure: a leave-one-out approach for TD [38] and a cross-validation one for HC [10]. Specifically, for the TD group, the training dataset of the SPT model was constructed for each examined subject using averaged angular velocity acquired during the first repetition of the nine remaining subjects. The angular velocity gathered from the second repetition, related to the examined subject, was used for the validation stage. The leave-one-out approach was repeated for all subjects, leaving one subject out of the training dataset in turn. Conversely, for the HC group the average of angular velocities gathered from the first repetition of all the healthy subjects was used to compute the training dataset of the SPT model. Successively, the second repetition of each subject was used for the validation stage.

### 2.5. Data Analysis

The generic examined classifier could be written as:

(2) TpmRωtc

where:

- pm is the chosen gait-phase model; it can be two phases (2P), four phases (4P) or six phases (6P);
- TR is the type of training used; it can be subject-specific training (SST) or standardized parameters training (SPT);
- tc represents the type of examined classifiers; it can be scalar classifier (SC) or distributed classifier (DC);
- ω is the angular velocity of the body segment used as input of the model, *i.e.,* foot (ft), shank (sh) or both at the same time (ftsh).

Since we have examined two training procedures, three classifiers based on three combinations of angular velocities, and three gait-phase models, a cluster of 18 classifiers was obtained for each subject of both groups and for each walking condition (Table 2).

**Table 2:** List of the all examined classifiers. SST is subject-specific training, SPT is standardized parameters training; 2P, 4P and 6P are the two-phase, the four-phase and the six-phase models; SC the scalar classifier and DC the distributed classifier; and ft the foot, sh the shank and ftsh the foot and the shank at the same time.

	SST			SPT		
**Two-phase model**	S2pSTftSC	S2pSTshSC	S2pSTftshDC	S2pPTftSC	S2pPTshSC	S2pPTftshDC
**Four-phase model**	S4pSTftSC	S4pSTshSC	S4pSTftshDC	S4pPTftSC	S4pPTshSC	S4pPTftshDC
**Six-phase model**	S6pSTftSC	S6pSTshSC	S6pSTftshDC	S6pPTftSC	S6pPTshSC	S6pPTftshDC

The performance of the proposed classifiers was evaluated by means of two indices: sensitivity, also addressed as true positive rate (TPR), and specificity, also addressed as true negative rate (TNR). The indices were calculated taking as a reference the state sequence obtained through the foot-switches [1,10]. We assumed the transition as the positive event and the non-transition as the negative event to detect. The phase transitions accordingly detected by classifier and reference signal were considered true positives; otherwise they were considered false positives. The non-transitions that were similarly detected by classifier and reference signal were considered true negatives, and those that were not, were considered false negatives.

From these considerations, TPR and TNR are defined as:

(3) $$TPR=\frac{True\;Positive}{True\;Positive+False\;Negative}$$

(4) $$TNR=\frac{True\;Negative}{False\;Positive+True\;Negative}$$

A tolerance window of 60 ms centered at each time step was applied to calculate both indices [1,10,39]. By means of TPR and the complement of TNR, which is called the false positive rate, we performed a Receiver Operating Characteristic (ROC) curve analysis, which is the well-accepted methodology for investigating the capability of a classifier in features classification [40].

As a global index of the capability of each classifier in the ROC space, we adopted the goodness index (G), already introduced in [13], as a complement to the Youden index introduced in [41]. G is expressed as:

(5) $$G=\sqrt{{\left(1-TPR\right)}^{2}+{\left(1-TNR\right)}^{2}}$$

Thus, G represents the Euclidean distance between the evaluated point in the ROC space and the point [0 1], which represents the classifier with a perfect performance in the ROC space. G can assume values between 0 and $\sqrt{2}$, and a classifier can be considered (i) optimum when G ≤ 0.25; (ii) good when 0.25 < G ≤ 0.70; (iii) random if G = 0.70, and (iv) bad if G > 0.70 [40]. The G index was computed for all the eighteen here examined classifiers, then means and standard deviations of G for all subjects and for each walking condition were calculated.

G values so obtained were tested for normality through the Shapiro-Wilk test [42]. Successively, two-way ANOVAs were performed on G data to find significant differences between the two training procedures and among gait-phase models. Statistical significance was set at 0.05. When significant differences were found, a Bonferroni’s test for multiple comparisons was performed. The software package SPSS (IBM-SPSS Inc., Armonk, NY, USA) was used.

The statistical power of the analysis was computed with software G*Power [43].

## 3. Results

**Figure 3:**
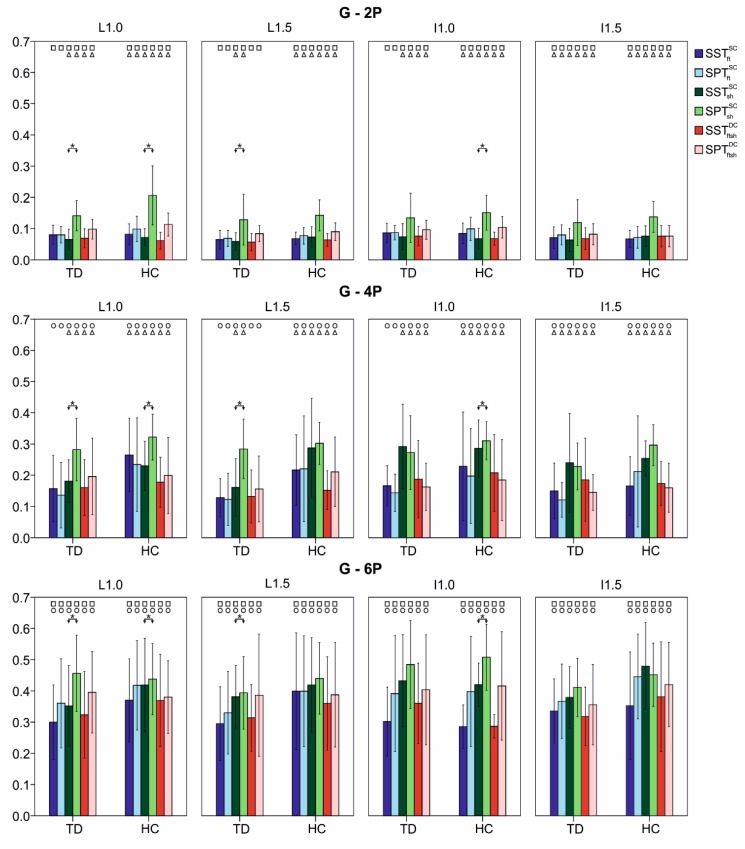
Goodness (G) mean values and standard deviations (error bars) for typically developed children (TD) and children with hemiplegia (HC) in the four walking conditions (L1.0, L1.5, I1.0, I1.5) computed with scalar and distributed classifiers and with the two training procedures (SST and SPT). Statistical difference between: (i) the SST and SPT procedures are marked with *; (ii) two-phase model (2P) and four-phase model (4P) with a triangle; (iii) 2P and six-phase model (6P) with a square and; (iv) 4P and 6P with a circle.

In Figure 3 the mean values and standard deviations of the Goodness index G for both TD and HC groups in the four walking conditions (L1.0, L1.5, I1.0 and I1.5) are reported, as a function of the model 2P, 4P and 6P.

All the examined classifiers presented mean values of G that were found to be in (i) the optimum range in 2P, (ii) the good/optimum range in 4P, and (iii) the good range in 6P. The standard deviations were found to be always less than 0.2, with higher values reached by classifiers applied on data gathered from HC group and relative to 6P model.

More specifically in the 2P model the best value of G was reached by S2pSTftSC for TD group (G = 0.06) and S2pSTftshDC for HC group (G = 0.06), both related to L1.5, while in both groups S2pPTftSC applied on data gathered by trial of I1.0 represented the worst classifier (TD: G = 0.20, HC: G = 0.21). Taking into account the 4P model, S4pPTftSC for L1.5 trial and S4pSTftshDC for I1.0 trial achieved the better value of G, for the TD group (G = 0.12) and HC group (G = 0.14), respectively. The lower value of G was, instead, obtained for both groups by S4pPTshSC in L1.0 (G = 0.32). As concerns the 6P model, G = 0.29 represented the best achieved value and it was obtained by S6pSTftSC for TD in L1.5 and by S6pSTftshDC for HC in I1.0. Conversely, S6pPTshSC achieved the lowest value (G = 0.51) for both groups in I1.0.

### Statistical Results

The outcomes of power statistical analysis showed a mean power value of about 85% for both independent variables, *i.e.* training procedures and gait-phase models, with a medium effect size (0.5) [44].

No significant interaction between training procedures and gait-phase models emerged for each walking condition, each group and each classifier (*p* > 0.1).

Comparing the SST and SPT procedures, the scalar classifier of the foot and the distributed classifier did not show statistical differences in each walking condition and for each group of subjects (*p* > 0.1); see blue and red histograms in Figure 3. As regards the scalar classifier of the shank (green histogram in Figure 3), differences between the two training procedures were found in L1.0 for both groups (TD: *p* = 0.001; HC: *p* = 0.002), L1.5 for TD (*p* = 0.006) and I1.0 for HC (*p* = 0.002).

Taking into account the effect of gait-phase model, statistical differences were found in all examined classifiers for both groups and in all walking conditions (*p* < 0.001). More precisely, from Bonferroni’s test for multiple comparison it emerged that the 6P model was always different from 4P and 2P (*p* < 0.001)—see square and circle symbol in Figure 3. Moreover, no statistical differences between 2P and 4P were found only in the TD group; more precisely, in the scalar classifier of foot in L1.0 (not entirely significant, *p* = 0.100), L1.5 (not extremely significant, *p* = 0.070) and I1.0 (not extremely significant, *p* = 0.060) and the distributed classifier in L1.5 (not extremely significant, *p* = 0.070). In the remaining cases, statistical differences were found between 2P and 4P (from weak significant to significant, 0.001 < *p* < 0.04); see triangle symbol in Figure 3.

## 4. Discussion

In this work we validated the inter-subject training procedure, called standardized parameter training (SPT), for the HMMs in the gait detection of typically developed children (TD) and children with hemiplegia (HC), with the aim of eliminating the subject-specific training stage. SPT is based on the estimation of the standardized walking gait of healthy subjects that represents the input of the training stage. This solution is needed to reduce (i) the time spent optimizing classifier parameters to the specific subject before the gait-phase recognition and (ii) the complexity of the measurement system. SPT demonstrated a significantly higher goodness index (G) values for the scalar classifier fed with shank data for all three gait models (2P, 4P, and 6P) and in both TD and HC groups. In particular, the reduction of performance was observed for all walking conditions except for inclined walking at 1.5 km/h (I1.5). Despite the increase of goodness values showed with the SPT procedure, G values remained in the same range of goodness exhibited with subject-specific training (SST). No significant variation of G was observed for the three gait-phase models, for the four walking conditions (L1.0, L1.5, I1.0 and I1.5) fed with foot angular velocity data. Similarly, no significant difference was found for distributed classifiers. These results encourage the use of the SPT procedure validated here to bypass the subject-specific training stage in pediatric subjects, when using both a distributed classifier fed with angular velocity data from foot and shank, as well as the scalar classifier of the foot. The found differences related to a scalar classifier (SC) fed with shank data are due to the greater inter-subject variability of segment angular velocity, observed in both groups, which implies a more difficult identification of the standardized parameter dataset. In the case of a scalar classifier of shank, the SPT procedure will lead to a performance decrement, even though the observed G values lie in the same range.

It is worth noting that the inter-subject procedure exhibits a better performance than the intra-subject one in the HC group, and only for the 4P model. This unexpected result, observable in both I1.0 and I1.5 for distributed classifiers and scalar classifiers of the foot, can be justified by the higher intra-subject variability of gait in patients with hemiplegia than the intra-subject variability in healthy children, confirmed by the higher standard deviation observable in gait data for the HC group. Thus in SPT, the use of a standardized dataset collected with healthy subjects, characterized by a higher similarity of the angular velocities among several strides, seems to lead to a more robust trained model than the one constructed with SST. Moreover, this finding confirms the outcomes discussed by Gorsic *et al.* in [29], where the authors stated that, in some conditions, the inter-subject procedure produced better performance than the intra-subject one.

Our findings, obtained by comparing SST with SPT, open the possibility of simplifying the overall preparation stage for automatic gait phase recognition. In the robotic rehabilitation field, indeed, the use of the SPT for phase recognition reduces the complexity of the sensor system of an active orthotic device by omitting foot-switch signals, and a higher usability of the device can be achieved by avoiding the time spent for the adaptation of the model parameters to the specific patient.

The comparison among the three gait-phase models here examined highlighted that a higher granularity of the gait partitioning leads to a lower performance of the classifiers. In fact, two-phase classifiers showed lower values of G, while the highest ones were obtained in a 6P model. Specifically, differences were observable among models for both TD and HC groups in all walking conditions, both using SST and SPT. This finding reflects the variability of the angular velocity waveforms during the different phases, as shown in Figure 2. From a comparative examination of 2P and 4P models, it emerged that the small difference between angular velocity patterns in flat foot (FF) and heel strike (HS) causes an increase of G values. In fact, when the discrimination of only two phases is required, FF and HS are included in the same phase, *i.e.*, SP. Furthermore, in the 6P model, the similar trend of angular velocity signal during loading response (LR), mid stance (MS) and terminal stance (TS) increase the difficulty in the state estimation for the HMM. Such a phenomenon was more evident in the pathological gait, confirming findings in [15], where authors assessed that LR was not always recognized by the HMM algorithm in subjects with movement disorders.

As a general conclusion about the gait-phase models, 2P and 4P should be preferred in applications where the reliability of the classifier is critical, such as in the implementation of a control system for lower limb exoskeletons. Conversely, a 6P model could be considered in exploratory applications, where a higher trade-off between granularity and reliability could be required.

No interaction between training procedures and gait-phase models emerged from the statistical analysis. This outcome highlights that differences among the gait-phase models are not dependent on the training procedure and vice versa, encouraging the application of the SPT procedure also to models of a granularity level higher than the maximum considered in the present study.

The scalar classifiers trained with SST showed non-relevant differences of performance regardless of the granularity of the model. More specifically, the scalar classifiers of the shank in most cases reached lower values of G with respect to the scalar classifiers of the foot. This result appears to be in disagreement with the findings reported in a previous study conducted by the authors [10], in which the scalar classifier of foot showed better performance. We argue that the reason for this disagreement could lie in the difference of the populations targeted in the two studies. Differently from [10], where healthy adults were enrolled, in the present study we recruited a cohort of subjects under 11 years old, which is considered a threshold age for the complete maturation of the ankle joint in terms of gait kinematics [30]. The non-mature behavior of the ankle joint of subjects in the TD group seems to imply a reduced angular activity; thus, the angular velocity signals of shank and foot are more similar than in adults. Such a consideration could be responsible for results obtained with the distributed classifier whose use does not improve the performance of the scalar classifiers. The expected higher performance of the distributed classifier was instead confirmed in patients with hemiplegia, where the goodness parameter reached lower values than the ones obtained by scalar classifiers. As a general conclusion, the use of a distributed classifier is useful when the waveforms of the two or more signals feeding the algorithm are not similar.

All above-mentioned results raised the possibility to design the sensor system of exoskeletons based only on IMUs without the use of footswitches, which present several critical issues: (i) their service life is shorter than inertial sensors; (ii) their robustness is compromised by the numerous and tiny leads; (iii) they require the design of a specific sole for each foot size; and (iv) an instrumented sole has to be designed even if the robotic device is used for rehabilitation of more proximal joints, such as the knee and hip, increasing the complexity of the device and decreasing its wearability.

## 5. Conclusions

In this paper we validated the inter-subject procedure, already proposed in literature for gait-phase detection for adults, to train scalar and distributed classifiers, based on a Hidden Markov Model (HMM), for phase detection in pediatric gait. The procedure, here validated on typically developed children and children with hemiplegia, consists in the identification of a standardized-parameter set obtained from angular velocity data. We compared the inter-subject procedure with an intra-subject one on three models of gait-phase partitioning (two, four and six phases) and with three classifiers (scalar classifier of the foot, scalar classifier of the shank, and distributed classifier). Our results demonstrated that the procedure represents a viable alternative to subject-specific training for the scalar classifier of the foot and the distributed classifier in gait detection of pediatric subjects. Instead, differences between the two examined procedures were found when the scalar classifier of the shank was examined, even though the two procedures reached goodness index (G) values in the same quality range. These findings raised the possibility of bypassing the time spent to tune classifier parameters to the specific subject gait pattern and, consequently, simplifying the sensor system of an exoskeleton without the use of additional sensors such as footswitches. From the comparison among different gait‑phase models it emerges that the performance decreases with the increase of model granularity. All the examined classifiers reached G values ranging from good in the six-phase model to optimum in two- and four-phase models. The distributed classifier revealed itself as a useful methodology to improve classification quality in children with hemiplegia.
